# Talking to Cows: Reactions to Different Auditory Stimuli During Gentle Human-Animal Interactions

**DOI:** 10.3389/fpsyg.2020.579346

**Published:** 2020-10-15

**Authors:** Annika Lange, Lisa Bauer, Andreas Futschik, Susanne Waiblinger, Stephanie Lürzel

**Affiliations:** ^1^Department for Farm Animals and Veterinary Public Health, Institute of Animal Welfare Science, University of Veterinary Medicine, Vienna, Austria; ^2^Department of Applied Statistics, Johannes Kepler University Linz, Linz, Austria

**Keywords:** cattle, animal welfare, human-animal communication, auditory perception, gentle talking, affective states, positive emotions, expressive behavior

## Abstract

The quality of the animal-human relationship and, consequently, the welfare of animals can be improved by gentle interactions such as stroking and talking. The perception of different stimuli during these interactions likely plays a key role in their emotional experience, but studies are scarce. During experiments, the standardization of verbal stimuli could be increased by using a recording. However, the use of a playback might influence the perception differently than “live” talking, which is closer to on-farm practice. Thus, we compared heifers' (*n* = 28) reactions to stroking while an experimenter was talking soothingly (“live”) or while a recording of the experimenter talking soothingly was played (“playback”). Each animal was tested three times per condition and each trial comprised three phases: pre-stimulus, stimulus (stroking and talking) and post-stimulus. In both conditions, similar phrases with positive content were spoken calmly, using long low-pitched vowels. All tests were video recorded and analyzed for behaviors associated with different affective states. Effects on the heifers' cardiac parameters were assessed using analysis of heart rate variability. Independently of the auditory stimuli, longer durations of neck stretching occurred during stroking, supporting our hypothesis of a positive perception of stroking. Observation of ear positions revealed longer durations of the “back up” position and less ear flicking and changes of ear positions during stroking. The predicted decrease in HR during stroking was not confirmed; instead we found a slightly increased mean HR during stroking with a subsequent decrease in HR, which was stronger after stroking with live talking. In combination with differences in HRV parameters, our findings suggest that live talking might have been more pleasurable to the animals and had a stronger relaxing effect than “playback.” The results regarding the effects of the degree of standardization of the stimulus on the variability of the data were inconclusive. We thus conclude that the use of recorded auditory stimuli to promote positive affective states during human-animal interactions in experimental settings is possible, but not necessarily preferable.

## Introduction

The welfare of animals is strongly influenced by the animals' perception and evaluation of their environment and the affective reactions induced by it (Veissier and Boissy, 2007). Humans constitute a substantial part of their environment, especially in farm animals. The way animals perceive humans and the quality of their interactions has a strong impact on their welfare (Boivin et al., 2003; Waiblinger, 2019). How an interaction is perceived by an animal can be influenced by the behavior shown by the human: characteristics of movements, tactile interactions and the use of voice all contribute to whether an interaction is experienced positively, neutrally or negatively (Waiblinger, 2017). While the perception of tactile stimulation has been investigated in cattle (Schmied et al., 2008; Lange et al., 2020), less is known about the effects of vocal stimulation (Waiblinger, 2017). Despite possible benefits of applying auditory stimuli in farm environments (Waiblinger, 2019), research on the effects of gentle vocal interactions on farm animals is scarce.

Cattle have highly developed auditory abilities: their hearing ranges from 23 Hz to 37 kHz (Heffner, 1998). Vocalizations are an integral part of their intraspecific communication (Kiley, 1972; Watts and Stookey, 2000; Green et al., 2019); for instance, in an affiliative context, cows direct low-frequency calls toward their calves (Padilla de la Torre et al., 2016). But cattle are also responsive to human vocalizations: calves can learn to be called by individual names (Murphey and Moura Duarte, 1983) and cows learn to follow specific calls to go to the milking parlor (Albright et al., 1966). They also seem to be sensitive to characteristics of voice reflecting the human's affective state: heifers showed a clear preference for handlers talking gently compared to handlers shouting at them (Pajor et al., 2003); however, visual signals might have influenced their choice in this experiment.

Low-pitched vocal interactions with drawn-out vowels are considered part of positive, friendly milker behavior (Waiblinger et al., 2002; Ivemeyer et al., 2011). Both in practice (e.g., Waiblinger et al., 2003; Hanna et al., 2006) and in research (e.g., Rushen et al., 1999; Schütz et al., 2012), gentle interactions with cattle often include gentle tactile stimulation in combination with talking in a gentle, soothing voice. However, it is difficult to standardize talking in the context of scientific experiments without introducing artificiality by repeatedly using the same phrases. Using playback of recordings facilitates the repeated presentation of auditory stimuli and might be useful for simplification of experimental designs (Watts and Stookey, 2000). There is evidence that calves recognize recorded samples of their mother's calls (Barfield et al., 1994), and the playback of recorded calls of calves stimulated milk production in cows (Pollock and Hurnik, 1978; McCowan et al., 2002) and lowered their heart rate (Zipp et al., 2014). The playback of a recording of gentle talking over a loudspeaker could increase standardization while retaining a natural speech melody. However, there are no studies that investigated if the use of speakers is equally effective as talking directly to cattle, as the animals might perceive the vocal stimulus differently. Recorded speech differs in frequency composition, harmonics and resonance from speech generated directly by a human (Howard and Angus, 2006), and losses in lower and higher frequencies are visible in sonographic recordings of recorded compared to live spoken voice commands (Fukuzawa et al., 2005). Another difference might be the loss of multimodal information when the auditory stimulus is produced artificially and presented via the single channel of a playback, excluding other multimodal components (Watts and Stookey, 2000). Furthermore, if one single recording is used for multiple experimenters to achieve increased standardization, the resulting mismatch between the broadcasted voice and the individual experimenter might disturb the animal, since studies show that domestic animal species such as horses can form cross-modal representations about familiar human individuals (Proops and McComb, 2012). In addition, talking in a gentle voice might also change the handler's affective state and body language, as vocalization, breathing and posture are closely related to the quality of sound produced (Partan, 2013), and that way might influence the animals' perception of the interactions and the resulting affective state.

To investigate the effects of human-animal interactions on the affective states of animals, different behavioral and physiological parameters can be measured (Mendl et al., 2010). The valence of animals' affective experience can be evaluated by observing their behavior (Dawkins, 2015; Kremer et al., 2020), including their facial expressions (for a review see Descovich et al., 2017). During social licking (Sato et al., 1991; Laister et al., 2011) and stroking by humans (Schmied et al., 2008) cattle often show neck stretching, a behavior interpreted as indicative of a positive experience. Additionally, recent studies suggest that ear positions and movements can be helpful in the assessment of affective states in cattle (e.g., Lambert and Carder, 2019; Lange et al., 2020). Other indicators for affective states are cardiac parameters, e.g., the HR of heifers accelerated when exposed to recordings of human shouting (Waynert et al., 1999). Heart rate (HR) is regulated by sympathetic and parasympathetic activity. Heart rate variability (HRV) parameters reveal more detailed information about sympathovagal balance and thus allow investigation of internal states of animals (von Borell et al., 2007).

We compared heifers' reactions to stroking while an experimenter was talking soothingly (“live”) or while a recording of an experimenter talking soothingly was played (“playback”). Even though earlier studies suggest that stroking in combination with auditory stimuli can elicit a positive, low-arousal state in cattle, this has not been shown for a stroking treatment with a playback auditory stimulus. We thus hypothesized that both forms of auditory stimulation in combination with stroking would lead to a positive, low-arousal state in the heifers; thus, we predicted a decrease of HR, an increase of HRV and an increase of behaviors indicating low arousal and positive valence. We expected some of these effects to last until shortly after stroking. Further, we hypothesized that live talking would elicit a more positive emotional state than talking played by a speaker. Finally, we hypothesized that the higher degree of standardization in the “playback” stimulus leads to lower variability in the data.

## Materials and Methods

### Animals, Housing and Management

The experiment was discussed and approved by the institutional ethics committee in accordance with the Good Scientific Practice guidelines and national legislation (project number ETK-02/04/2017).

The study was performed with 28 heifers (27 Austrian Simmental, one Austrian Simmental × Brown Swiss) on the young stock farm of the University of Veterinary Medicine, Vienna (Rehgras, Furth an der Triesting, Austria) between May and November 2017. As we aimed to investigate positive emotions during human–animal interactions, a generally positive perception of close human contact was a prerequisite. Based on their positive animal-human relationship, we pre-selected 32 heifers. Twenty-eight of these animals were later used for the tests. The heifers' age ranged from 7 to 24 months. According to their age, two groups of 16 animals were formed. Housing, feed and general treatment was the same for both groups, which were kept mainly on pasture. Only during poor weather conditions and for testing the animals were brought into deep-litter pens with adjoining outdoor runs, where they were fed hay and concentrate. Water and mineral blocks were provided ad libitum.

The animals were carefully habituated to the camera (Sony HDR-CX730,Weybridge, UK) and HRV equipment (Polar Electro Oy, Kempele, Finland) as well as the experimenters (both female, green overalls; A: brown hair, 1.63 m; B: brown-reddish hair, 1.70 m), the loudspeakers (Denon Envaya mini™ DSB-100, Kawasaki, Japan; fixed to the strokers' chest, but not playing sound) and the stroking procedure, until it was possible to equip the free-moving heifers with the HRV girths and stroke the animals for 3 min without them walking away or showing any visible signs of unease. Animals were considered fully habituated when a full 9-min trial (see Section Experimental Procedure, no vocal stimulation) could be performed on them while they were lying without inducing any avoidance reactions. For further details of the selection and habituation process, see Lange et al. (2020).

### Experimental Design

We applied a crossover design, i.e., each animal acted as its own control and was thus subjected to both treatments. To ensure robustness of the data, each animal experienced each treatment three times in an alternating pattern, i.e., in a total of six trials (trial numbers 1–6). Each trial consisted of three phases of 180 s (3 min) each: (1) pre-stimulus (PRE), where the experimenter stood next to the animal so that baseline values could be recorded; (2) stimulus (STIM), with the experimenter stroking the ventral neck while talking in a gentle voice (“live”) or while a recording of the experimenter talking in a gentle voice was played (“playback”), and (3) post-stimulus (POST), where the experimenter was standing next to the animal again so that possible carry-over effects could be observed. Approximately half of the animals started with the “live” auditory stimulus, the other half with the “playback” stimulus. The experimenters aimed to balance the order of the treatments over each testing day, but complete balancing was not always possible.

### Experimental Procedure

#### General Procedure

All trials were carried out in a deep-litter barn of 182 m^2^ (min. 11 m^2^/animal), which was familiar to all animals. Each animal was prepared and equipped for HRV measurement (POLAR® horse trainer transmitters and S810i monitors, Polar Elektro Oy, Kempele, Finland) by thoroughly wetting the coat and applying ultrasound gel at electrode sites, before using elastic girths to fix the electrodes and transmitters to the chest. The transmitters were protected by a second girth with a sewn-on pocket to contain the monitor. All trials were conducted on lying animals during resting phases to minimize the influence of physical activity on cardiac parameters. Before starting a trial, the handler (i.e., stroker) started a POLAR® monitor and placed it in the pocket of the girth. When an animal had been lying for at least 5 min, the camera operator assumed a position ~2 m from the heifer with the camera approximately at the height of the heifer's eyes, filming the head/neck region from the heifer's left side with special focus on the left eye and ear. The stroker assumed a standing position next to the animal's left shoulder and started the trial. She wore rubber gloves with a rough surface and, when the STIM phase started, applied a constant, previously practiced pressure while stroking at a frequency of 40–60 strokes/min (Schmied et al., 2008). The loudspeaker was hanging around the strokers' neck and fixed to the stroker's chest. A trial was completed after 9 min or aborted earlier at the occurrence of an event likely to influence the animal's emotional or physiological state, e.g., standing up, falling asleep or social interactions (Lange et al., 2020). If a trial was stopped, the experimenters waited for at least 1 h before testing the animal again.

#### Auditory Stimuli

During the stimulus phase, all animals experienced tactile stimulation on the ventral neck as described in Lange et al. (2020). Additionally, they were exposed to different auditory stimuli. In the “live” condition, the stroker talked directly to the animals in a gentle voice as in previous studies (Lürzel et al., 2015b, 2016), using phrases with positive content (in German) that were spoken calmly, with long low-pitched vowels and a decrease in pitch toward the end of the words or phrases. For the “playback” condition, a sample of Experimenter A talking in a gentle voice in the same way as in the “live” condition was recorded in WAV format via a digital voice recorder (Linear PCM Recorder LS-3, Olympus, Japan). It was integrated into an audio file (see Supplementary Data 1) that was played via an MP3 player (SanDisk Clip Sport MP3 Player, SanDisk Corporation, Milpitas, USA) connected to the loudspeaker fixed to the strokers' chest. The volume of the loudspeaker was adapted (using the Smartphone Android App SoundMeter) to the volume of the experimenter talking before each sequence of trials, as the experimenter adjusted the volume of her voice to the surroundings (e.g., wind, farm work). We determined an average volume of 35–47 dB per day, while staying under a maximum level of 70 dB. To assess the acoustic qualities of our recording we used the free acoustic analysis software Praat (Boersma and Weenink, 2020). The mean pitch was 190.7 Hz (± 43.4 Hz standard deviation), which is lower than the mean pitch of a sample that was described as a soothing voice cue (236.2 Hz) in contrast to a harsh voice cue (322.1 Hz) (Heleski et al., 2015).

While the experimenter was stroking the animals continuously during the 3 min of the STIM phase, the vocal stimulus was only present in the first and last minute. In both conditions, spoken signals in the audio file announced the start and end of these 1-min periods as well as of the phases. Between the phases, there were 10-s breaks to allow the stroker to assume or leave the stroking position. Possible effects of the loudspeaker itself were thus present in both conditions and the auditory stimulus of the playback was as similar as possible to the “live” condition with respect to duration of speech. Two persons conducted the experiments; one stroked the animals, the other filmed the treatment. In two thirds of the trials the stroking treatment was performed by Experimenter A and in one third by Experimenter B, in a semi-randomized order.

### Behavioral Observations

All trials were video recorded and the behavior was analyzed with the coding software Solomon Coder (version: beta 17.03.22, András Péter, Budapest, Hungary), using focal animal sampling and continuous recording (Martin and Bateson, 2007). The observer was blinded to the test condition as the head of the stroking person was covered on the screen during coding, so that possible lip movements were not visible. The observer recorded ear and head positions and movements as well as other behavior according to an ethogram (Table 1; for photographs of ear positions, see Supplementary Figures 1, 2). To determine the intra-observer reliability, ten 2-min video sequences were chosen from videos not used for further analyses and coded twice. Cohen's kappa for ear postures was 0.61, for eye aperture 0.63 and for the head postures 0.71. Cohen's kappa for rumination and lying position was 1 and for miscellaneous behaviors 0.64.

**Table 1:** Ethogram (Lange et al., 2020).

**Category**	**Behavior^a^**	**Definition**
Inactive ear posture^b^	Ear hanging	The ear loosely hangs downwards (referring to the ground). There is no visible muscle tension, leading often to a slightly bouncing movement when the position is assumed.
Active ear postures^b^^,^ ^c^	Back up	The ear is held behind and above the latero-lateral axis.
	Back center	The ear is held behind and at the same height as the latero-lateral axis.
	Back down	The ear is held behind and below the latero-lateral axis.
	Center up	The ear is held perpendicular to the head and above the latero-lateral axis.
	Center	The ear is held perpendicular to the head along the latero-lateral axis.
	Center down	The ear is held perpendicular to the head and below the latero-lateral axis.
	Forward up	The ear is held in front of and above the latero-lateral axis.
	Forward center	The ear is held in front of and at the same height as the latero-lateral axis.
	Forward down	The ear is held in front of and below the latero-lateral axis.
	Ear flicking	The ear is quickly (within max. 0.5 s) moved back and forth at least once. The behavior is coded until one of the other ear postures is clearly visible again. The residual movement after the active movement is still part of ear flicking.
Head/neck postures	Held without touching	The head is actively held up and does not touch the stroker.
	Held with touching	The head is actively held up and touches the stroker.
	Rest head without touching	The heifer does not actively carry the head's weight. The heifer's head is in contact with the ground, barn equipment, another animal or with the heifer's leg(s). The heifer's head is not in contact with the stroker.
	Rest head with touching	The heifer does not actively carry the head's weight. The heifer's head is lying on the ground, barn equipment, another animal or the heifer's leg(s) while being in contact with the stroker, or it is lying on the stroker's leg.
	Head shaking/tossing	Successive quick movements of the head. The movements can be rotational or up and down.
	Neck stretching	Positioning neck and head actively in an outstretched line, either up, down, or forward.
Eyes^d^	Open	The iris is at least partly visible.
	Closed	The iris is not visible at all for longer than 0.5 s.
	Not visible	Neither eye is visible.
Miscellaneous	Rubbing the stroker	The heifer touches the stroker and moves the touching body part while in contact with the experimenter. The behavior ends when the contact between the heifer and the person is interrupted for at least 3 s.
	Rubbing	The heifer moves the head/neck region while in contact with the ground or barn equipment. The behavior ends when the contact between the heifer's head/neck region and the ground/equipment has ended.
	Nose close	The heifer moves her muzzle toward the stroker within a range of 5 cm. The behavior ends when the heifer's nose does not point toward the stroker anymore, leaves the range of 5 cm or if another behavior of the “miscellaneous” category starts.
	Licking the stroker	The heifer's tongue touches the stroker at least once. The behavior ends when the heifer's tongue does not touch the stroker again within 3 s.
	Ruminating	The heifer's jaw moves regularly sideways with a frequency of about one movement per second. This movement is recorded as rumination if it occurs in a series of at least five movements (which may start before and end after the observation). Rumination ends when the jaw movement is paused for more than 10 s.
Calculated measures	Contact	The time in which the heifer's head and neck area was in contact with the stroker. Sum of durations of “rest head with touching”, “held with touching”, “nose close”, “rubbing experimenter” and “licking experimenter”, not including contact established by stroking.
	Resting head	Sum of durations of “rest head with touching” and “rest head without touching”.
	Ear low	The sum of the durations of the ear hanging or held below the latero-lateral axis (“hanging” + “back down” + “center down” + “forward down”).
	Changes of ear positions	Sum of the frequencies of different ear positions per trial minus 1.

a *All behaviors were coded as durations, except changes of ear positions (count data)*.

b *The left ear was recorded; if it was not visible, the right ear was recorded*.

c *The latero-lateral axis refers to an imaginary line between the bases of the ears. “Behind” means the ear is pointing toward the back of the head, “in front” refers to the rostral end of the head, “above” describes the ear pointing dorsally and “below” pointing ventrally. If the observed ear was moved by the experimenter, the position before the movement was recorded until the next unambiguous ear posture was assumed*.

d *The left eye was recorded; if it was not visible, the right eye was recorded*.

### Heart Rate Measurements

Inter-beat intervals were error-corrected and processed according to Hagen et al. (2005) using the Polar Precision Performance Software, version 4.03.050 (Polar Electro Oy, Kempele, Finland), and HR and HRV parameters were calculated using Kubios, version 2.0 (Biosignal Analysis and Medical Imaging Group, Department of Applied Physics, University of Eastern Finland, Kuopio, Finland). To account for the respiratory rate, frequency bands were set to 0.04–0.2 Hz for the low frequency band and 0.2–0.58 Hz for the high frequency band (von Borell et al., 2007). The following parameters were analyzed statistically: mean heart rate (*HR*); time domain: standard deviation of the inter-beat intervals (*SDNN*) and square root of the mean squared differences of successive inter-beat intervals (*RMSSD*), and the ratio of *RMSSD* and *SDNN* (*RMSSD*/*SDNN*); frequency domain (using fast Fourier transform): normalized powers of high (*HF*) and low frequency (*LF*), and the ratio of *LF* and *HF* (*LF*/*HF*).

### Statistical Analysis

#### Behavioral Data

We used the software package R, version 3.5.2 (R Core Team, 2019). The durations of behaviors that occurred often enough to be suitable for analysis were transformed to proportions by dividing them by the total time during which they could be observed. To account for the fact that the ear positions are mutually exclusive and their proportions always amount to one, we tried to fit a compositional model but the large amount of zeros led to convergence problems. Therefore, we selected the three ear positions that were observed often enough for statistical analysis. They were analyzed using generalized linear mixed models (GLMMs) (Baayen, 2008) with a beta error structure and logit link function (McCullagh and Nelder, 1989; Bolker, 2008) using the package “glmmTMB,” version 0.2.3 (Brooks et al., 2017). Because values of the responses being exactly 0 or 1 can lead to infinite point probabilities in beta distributions, the response variables were transformed according to (y × (n – 1) + 0.5)/n, where y is the original response and n the number of observations (Smithson and Verkuilen, 2006), resulting in regular small shifts of the values away from 0 and 1 (e.g., for *n* = 534, 0 becomes 0.00094, 1 becomes 0.99906).

*Ear hanging* and the other downward ear positions did not occur often enough to be evaluated statistically on their own [median duration in s (min–max): *hanging* 0 (0–155)]. Thus, we calculated the variable *low ear* by summing up the durations of downward ear positions (*hanging* + *back down* + *center down* + *forward down*; summed up to *low*). The result of *low ear* was still dominated by zeros, causing difficulties with the beta error distribution; therefore, it was dichotomized (occurrence: yes/no) and analyzed using a GLMM with a binomial structure and logit link function. The behavior *changes of ear position* was calculated by summarizing the frequency of different ear positions and subtracting 1 (for the initial ear position), and analyzed using a GLMM based on the negative-binomial distribution with a log link function. A minimum of three observations per condition per animal were included in statistical analyses. If additional tests were performed due to technical problems in HR(V) data collection, up to four tests per condition could be included (9 cases), which resulted in a sample size for models of 534 measures in total made for 28 individuals in a total of 178 trials with 3 phases each. For all full models, fixed effects were treatment (factor with two levels: live, playback), phase (factor with three levels: PRE, STIM, POST) and their interaction, and individual as well as trial ID (trial number nested in individual) as random effects. Trial ID was included as a random effect to account for the fact that each trial consisted of three phases and thus contributed three data points, where it seemed plausible to assume that there was random variation between the trials. We included random slopes within individual for trial number (to account for possible changes caused by treatment repetition), treatment and phase to allow their effects to vary between individuals (Barr et al., 2013). To address the issue of cryptic multiple testing (Forstmeier and Schielzeth, 2011), we compared each full model with a respective null model that lacked the variables of interest (phase and the interaction of phase and treatment) but was otherwise identical. We used a likelihood ratio test (R function “anova”) for these comparisons. The significance of the individual independent variables was determined by dropping them one at a time and using a likelihood ratio test to compare the resulting models to the full model (Barr et al., 2013). Values of *p* ≤ 0.05 are referred to as significant, and 0.05 < *p* ≤ 0.1 as a trend (Stoehr, 1999). If the full–null model comparison was, or tended to be, significant and the interaction was non-significant, the interactions were removed from the models and reduced models were fitted to investigate the main effect of phase. Main effects of treatment were not tested, as they were not of interest.

As stated above, the mismatch between the broadcasted voice and the individual experimenter stroking the animal, as was the case when experimenter B was stroking during the playback of the voice of experimenter A, could drive the results regarding the interaction between condition and phase. If this were the case, one would expect the pattern of this interaction to depend on whether the mismatch was present or not. To address this question, we fitted one model in addition to each full model. This model included the three-way interaction between phase, treatment and presence of the mismatch and all terms comprised therein (and a random slope of presence of the mismatch), but was otherwise identical to the respective full model. Subsequently, we compared this model to a reduced model lacking the three-way-interaction but otherwise being identical, again using a likelihood ratio test (R function “anova”). If this comparison reveals significance, it indicates that the effects of condition and phase were indeed driven by the mismatch. In the case of the model for the behavior *neck stretching*, the reduced model did not converge, but we inspected the coefficients of the full model to reveal possible effects of the mismatched experimenter/voice combination on the duration of *neck stretching*. We found no evidence for significant effects of the mismatch between the broadcasted voice and the individual experimenter stroking the animal for any of the behaviors (*neck stretching z* = 0.534, *p* = 0.593 (full model coefficients); *contact* χ^2^ = 0.223, df = 2, *p* = 0.895; *eye closed* χ^2^ = 0.025, df = 2, *p* = 0.988; *head resting* χ^2^ = 0.451, df = 2, *p* = 0.798; *ear flicking* χ^2^ = 0.916, df = 2, *p* = 0.632; *changes of ear position* χ^2^ = 0.522, df = 2, *p* = 0.770; *back up* χ^2^ = 0.077, df = 2, *p* = 0.962; *back center* χ^2^ = 2.746, df = *p* = 0.253; *forward up* χ^2^ = 0.937, df = 2, *p* = 0.626; *ear low* χ^2^ = 0.684, df = 2, *p* = 0.710). Hence, we report results of the models not including the mismatch.

Since the “playback” stimulus had a higher degree of standardization than the “live” stimulus, it seemed plausible that the variation in a given behavior would be smaller in the “playback” treatment than in the “live” treatment. We explicitly estimated this potential effect by modeling the precision parameter of the response as a function of treatment in each model (Lange et al., 2020). With a higher degree of standardization in “playback” stroking, we expected smaller variation in behaviors, and thus, larger estimated precision parameters. For the models where we found overdispersion (*neck stretching, changes of ear positions, contact, head resting* and *forward up*), we corrected standard errors and *p*-values based on Wald's z-approximation (Field, 2005); therefore no degrees of freedom are reported and χ^2^s were replaced by z-values (Gelman and Hill, 2006). We determined 95% confidence limits using the function “simulate.glmmTMB” of the “glmmTMB” package. We assessed the model stability by comparing the estimates of models based on the full dataset with estimates of models fitted to subsets where the levels of each random effect were dropped one at a time (Nieuwenhuis et al., 2012). This revealed a fairly good stability of the models.

For graphical depiction, we used the R packages “ggplot2” (Wickham, 2016) and “cowplot” (Wilke, 2019). Data were depicted as boxplots for each treatment and phase, using the mean values of behaviors per animal (averaged across the three trials per treatment). The bold line corresponds to the median; the lower and upper lines of the box to the first and third quartile, respectively; and the whiskers correspond to the lowest and highest values that were still within 1.5 × interquartile range from the margins of the box. Outliers (all values outside of 1.5 × interquartile range) are depicted as circles.

#### Cardiac Data

Due to technical problems during HRV recording (i.e., >5% of errors per minute), we obtained a sample size of 26 animals, which resulted in 176 total measures as sample size for models. Because of an insufficient number of recordings with experimenter B, only recordings of tests where experimenter A stroked the animals were used for HRV analysis. Cardiac variables were analyzed using linear mixed models (LMMs) with the package “lme4” (Bates et al., 2015), including treatment, phase and their interaction, age (d), time of day, *HR* (unless it was the response variable) and duration of rumination (s) as fixed effects. Heart rate was included as a fixed effect because it is often strongly correlated to HRV indicators (Zaza and Lombardi, 2001; Monfredi et al., 2014; Sacha, 2014; McCraty and Shaffer, 2015). While HR is often regarded as an indicator of arousal (Zebunke et al., 2013; Briefer et al., 2015; Travain et al., 2016; Lambert and Carder, 2019), HRV might also provide information on valence (Boissy et al., 2007). By correcting for *HR* in the models, the results represent the influence of the other independent variables (mainly the interaction of treatment and phase) on HRV parameters independently of their influence on *HR*, allowing conclusions in addition to those that can be drawn from *HR*. To account for the cyclical nature of circadian rhythms that influence HRV (Hagen et al., 2005; Kovács et al., 2016), we modeled time of day turning time into radians: first we transformed time to decimal numbers by summarizing hours, minutes divided by 60 and seconds divided by 3,600. The result was multiplied with 2 × π and divided by 24, and the resulting variable was included together with its sine and cosine into the model (Stolwijk et al., 1999). The individual and trial number nested in individual were considered as random effects. We included random intercepts and random slopes within individual for trial number (to account for possible effects of treatment repetition), treatment and phase to allow their effects to vary between individuals. Where possible, we also included estimates of the correlations between the random intercept and slopes into the model (Barr et al., 2013). However, for the response variables *SDNN* and *LF*, the models including the correlations did not converge and we dropped the correlation estimates from the model. We then proceeded in the same way as described above: we fitted a null model that lacked the variables of interest (phase and the interaction of phase and treatment), and if the full-null model comparison revealed significant differences and the interaction was non-significant, it was removed from the model and reduced models were fitted to test for the significance of the main effect of phase.

## Results

### Behavior During Gentle Interactions

**Figure 1:**
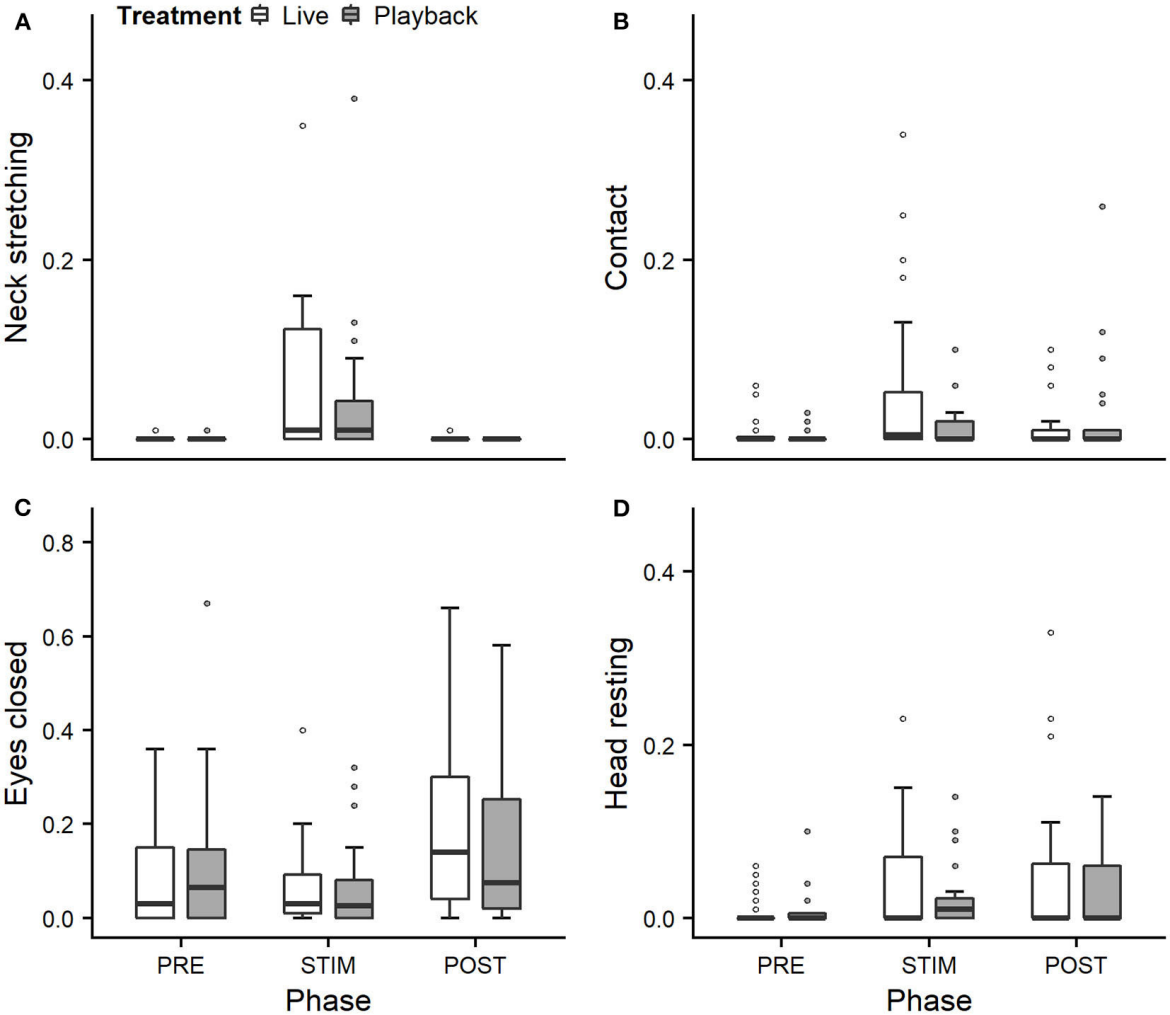
Mean durations (as a proportion of the total time observed) of *neck stretching* **(A)**, *contact* **(B)**, *eyes closed* **(C)**, and *head resting* **(D)** of heifers (*n* = 28) during the experimental trials. Means were calculated across the three trials per treatment and are depicted according to the treatment used (white = “live,” dark gray = “playback”) and phase (PRE = pre-stimulus, STIM = stimulus, POST = post-stimulus). Statistics for GLMMs: significant main effect of phase for *neck stretching* **(A)**, *p* < 0.05. Note that the y-axis scale varies to allow for sufficient resolution for rare behaviors.

**Figure 2:**
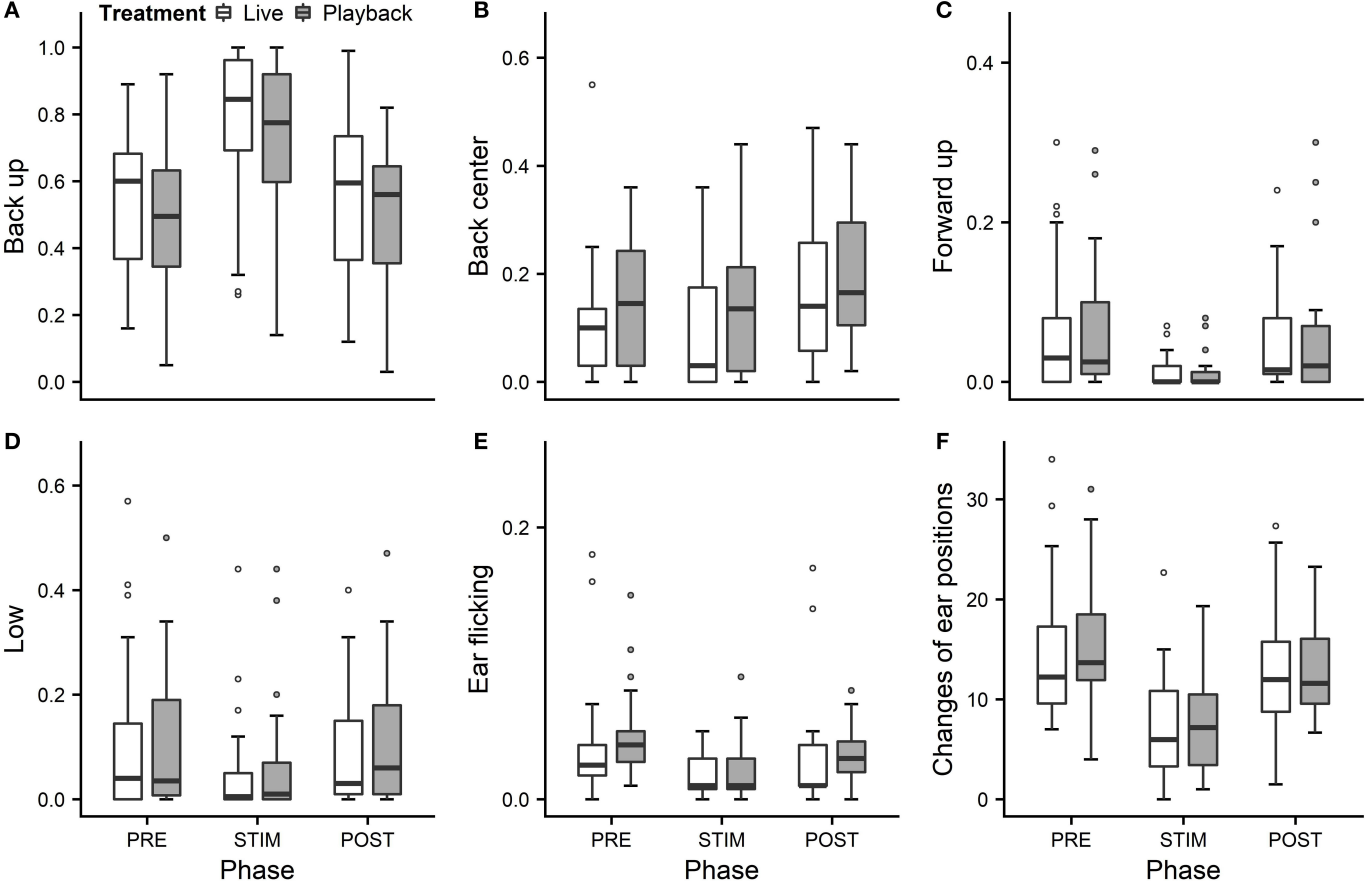
Mean durations of ear positions **(A–D)** and ear flicking **(E)** as a proportion of the total time observed and **(F)** mean number of changes of ear positions of heifers (*n* = 28) during the experimental trials. Means were calculated across the three trials per treatment and are depicted according to the treatment used (white = “live,” dark gray = “playback”) and phase (PRE = pre-stimulus, STIM = stimulus, POST = post-stimulus). Statistics for GLMMs: significant main effect of phase for *back up* **(A)**, *back center* **(B)**, *ear low* **(D)**, *ear flicking* **(E)**, and *changes of ear positions* **(F)**, *p* < 0.05; and trend for *forward up* **(C)**, *p* < 0.1. Note that the y-axis scale varies to allow for sufficient resolution for rare behaviors.

We statistically analyzed *neck stretching* (median duration in s; min–max: 0; 0–112), *contact* (0; 0–175), *eye closed* (0; 0–180) and *head resting* (0; 0–180) (Figure 1); the ear positions *back up* (124; 0–180), *back center* (8; 0–180), *forward up* (0; 0–164), *ear low* (0; 0–169); and the ear movements *ear flicking* (1; 0–76) and *changes of ear position* (9; 0–63) (Figure 2).

Full and null models differed significantly for the response variables *neck stretching* (Figure 1; GLMM: χ^2^ = 10.811, df = 4, *p* = 0.029), *ear flicking* (Figure 2; χ^2^ = 32.426, df = 4, *p* < 0.001) and *changes of ear position* (Figure 2; χ^2^ = 35.907, df = 4, *p* < 0.001) as well as for all the tested ear positions except for *forward up* (Figure 2; *back up*: χ^2^ = 31.371, df = 4, *p* < 0.001; *back center*: χ^2^ = 13.613, df = 4, *p* = 0.009; *ear low*: χ^2^ = 19.758, df = 4, *p* = 0.001). The full–null model comparisons revealed a statistical tendency toward a difference for *forward up* (Figure 2; χ^2^ = 9.332, df = 4, *p* = 0.053) and no significant difference for *contact* (Figure 1; χ^2^ = 2.067, df = 4, *p* = 0.723), *head resting* (Figure 1; χ^2^ = 2.024, df = 4, *p* = 0.731) and *eyes closed* (Figure 1; χ^2^ = 6.113, df = 4, *p* = 0.191).

As the interaction of phase and treatment was not significant for any of the behaviors we analyzed, effects of the phase were not influenced by the type of auditory stimulus used in the treatment. However, independently of which treatment was used, the phase had a significant effect on several of the behaviors. The reduced models revealed a significant main effect of phase for *neck stretching* (z = 2.594, *p* = 0.009), *ear flicking* (χ^2^ = 32.520, df = 2, *p* < 0.001) and *changes of ear position* (χ^2^ = 31.526, df = 2, *p* < 0.001): while the durations of *neck stretching* increased during STIM (Figure 1), the durations of *ear flicking* and the numbers of *changes of ear position* decreased (Figure 2). Phase also had a significant effect on the ear positions *back up* (χ^2^ = 30.705, df = 2, *p* < 0.001), *back center* (χ^2^ = 13.500, df = 2, *p* = 0.001), *forward up* (*z* = −0.216, *p* = 0.027), and *ear low* (χ^2^ = 19.094, df = 2, *p* < 0.001): during STIM, the durations of *back up* increased significantly, whereas the durations of the other tested ear positions decreased (Figure 2).

The variability was significantly smaller in the “playback” treatment for *neck stretching* (χ^2^ = 16.177, df = 1, *p* < 0.001) and *contact* (χ^2^ = 4.321, df = 1, *p* < 0.001), but higher for the ear position *back center* (χ^2^ = 10.273, df = 1, *p* < 0.001). It did not differ significantly for the other behaviors. For statistical details, including model coefficients, standard errors and confidence intervals, see Supplementary Material (Supplementary Table 1).

The number of tests aborted because of heifers standing up during STR without any obvious reason (e.g., being chased up) was higher in the “playback” condition (*n* = 13) than the “live” condition (n = 6) but did not differ significantly (χ^2^ = 2.3, df = 1, *p* = 0.127).

### Cardiac Data

Full and null models differed significantly for the response variables *HR* (LMM: *HR*: χ^2^ = 26.688, df = 4, *p* < 0.001), *SDNN* (χ^2^ = 13.185, df = 4, *p* = 0.010), *RMSSD*/*SDNN* (χ^2^ = 13.091, df = 4, *p* = 0.011) and *HF* (χ^2^ = 12.272, df = 4, *p* = 0.015). The full–null model comparison revealed no significant difference for *RMSSD* (χ^2^ = 2.933, df = 4, *p* = 0.569), *LF* (χ^2^ = 0.645, df = 4, *p* = 0.958) and *LF*/*HF* (χ^2^ = 2.784, df = 4, *p* = 0.595).

**Figure 3:**
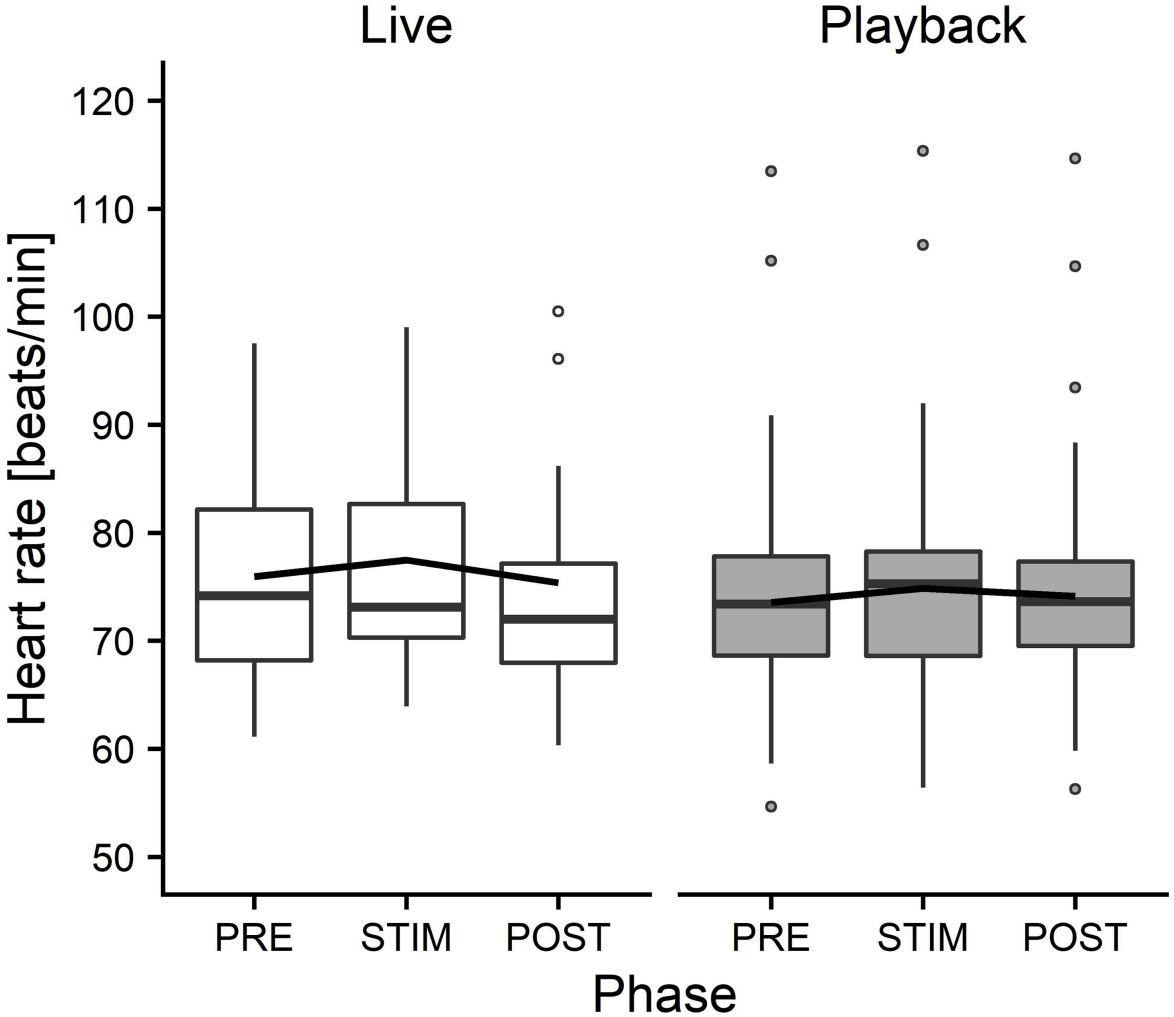
Means of heart rate of heifers (*n* = 26), calculated across the three trials per treatment and depicted according to treatment (white = “live,” gray = “playback”) and phase (PRE = pre-stimulus, STIM = stimulus, POST = post-stimulus). The black line indicates the estimated means of the models. Statistics for LMM: significant interaction of condition and phase, *p* < 0.05.

**Figure 4:**
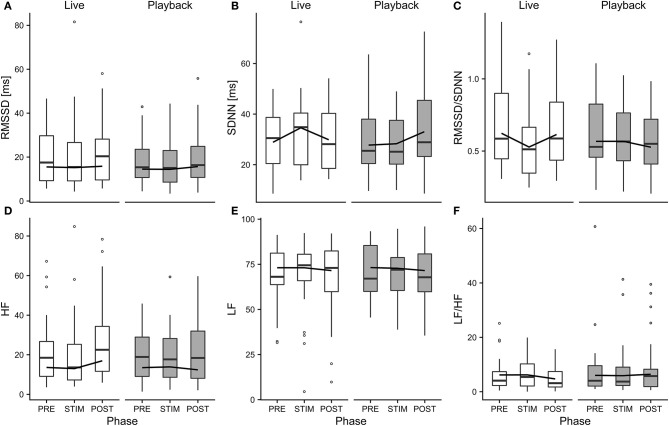
Means of heart rate variability parameters *RMSSD* **(A)**, *SDNN* **(B)**, *RMSSD/SDNN* **(C)**, *HF* **(D)**, *LF* **(E)**, and LF/*HF* **(F)** of heifers (*n* = 26), calculated across the three trials per treatment and depicted according to treatment (white = “live,” gray = “playback”) and phase (PRE = pre-stimulus, STIM = stimulus, POST = post-stimulus). The black line indicates the estimated means of the models. Statistics for LMMs: significant interaction of condition and phase for *SDNN* **(B)***, RMSSD/SDNN* **(C)**, *HF* **(D)**, *p* < 0.05.

The interaction of phase and treatment was significant for all cardiac parameters with a significant full-null model comparison (Supplementary Table 2, *HR*: χ^2^ = 9.917, df = 2, *p* = 0.007; *SDNN*: χ^2^ = 8.738, df = 2, *p* = 0.013; *HF*: χ^2^ = 7.657, df = 2, *p* = 0.022; *RMSSD*/*SDNN*: χ^2^ = 8.378, df = 2, *p* = 0.015). Whereas *HR* increased slightly during stroking in both conditions, it decreased more strongly in the “live” condition after the treatment (Figure 3). There was a distinct increase in SDNN during STIM in the “live” condition, followed by a decrease in POST, whereas the strongest increase in the “playback” condition took place in POST. *RMSSD*/*SDNN* mirrored this pattern: in “live” it decreased during STIM, increasing again in POST, and in “playback” it decreased during POST. *HF* increased by nearly 30% during POST of the “live” condition whereas it decreased during POST in “playback” (Figure 4). The models revealed a significant negative effect of *HR* on all the HRV parameters except *LF* and *LF/HF*, where it had a significant positive effect (see Supplementary Table 2).

## Discussion

We compared the reactions of heifers to stroking while applying two different auditory stimuli: the stroker talking directly to the animals in a gentle voice or a recording of the stroker's talking. We found behavioral and physiological indications of a positive perception of the interactions for both auditory stimuli. While the behavioral reactions to gentle interactions did not differ statistically, some of the cardiac parameters indicated differences between the auditory stimuli, also shortly after the presentation of the stimulus had ended.

### Perception of Each Treatment

Both treatments led to changes in behavior during the STIM phase that indicate a positive perception: During stroking, the heifers showed significantly longer durations of *neck stretching*, a behavior shown during intraspecific social grooming (Sambraus, 1969; Reinhardt et al., 1986; Schmied et al., 2005), which is often actively solicited, and stroking by humans (Waiblinger et al., 2004; Schmied et al., 2008; Lürzel et al., 2015a). It is interpreted a sign of enjoyment, and it can thus be assumed that the situation is perceived as positive.

In a previous, similar experiment (Lange et al., 2020), we observed decreases of *ear flicking* and *changes of ear position* during stroking with no auditory stimuli. The present study confirms this pattern. The animals showed less *ear flicking* during STIM than PRE, a behavior mostly associated with negative affective states, such as pain after dehorning (Heinrich et al., 2010; Neave et al., 2013) or reactions to insect attacks (Mooring et al., 2007).

During STIM, the animals also changed the positions of their ears less often than in PRE. Frequencies of *changes of ear positions* were lower in sheep feeding (Reefmann et al., 2009a) or voluntarily being groomed by a human (Reefmann et al., 2009b) than during separation from the herd. In contrast, dairy cows showed an increased frequency of *changes of ear positions* during stroking compared to before or after (Proctor and Carder, 2014), which might however have been caused by small differences in experimental design, such as the stroker approaching at the beginning of the stroking phase. In contrast, the decrease in *changes of ear positions* and *ear flicking* during stroking in the current as well as in our previous study (Lange et al., 2020) indicates an association of a reduction of these behaviors with a positive, low-arousal state also in cattle.

However, for some of the behaviors we expected to indicate affective states, the treatment did not lead to significant differences: previously observed effects of stroking (Lange et al., 2020) on the duration of the animal *resting* its *head* and the time spent in *contact* with the experimenter were not confirmed in this study. These findings might be connected with the auditory stimulus, which might keep the animal comparatively more attentive to a certain degree and thus limit the intensity of the relaxation.

In an attempt to reflect the continuous nature of ear positions, we recorded nine different positions along the vertical and the horizontal axis: *back up, back center, back down, center up, center, center down, forward up, forward center* and *forward down*, plus *ear hanging*. During stroking, durations of the *back up* position increased significantly, while durations of *forward up* and *ear low* decreased, mostly in line with our previous experiment (Lange et al., 2020). The tendency toward decreased durations of *forward up* might indicate lowered vigilance (Boissy and Dumont, 2002), which is associated with less fear (Welp et al., 2004), and could corroborate the hypothesis that stroking induces positive low-arousal states.

We predicted to find longer durations of *ear low* during stroking, because *low ear* positions, including *ear hanging*, were associated with low-arousal, positive affective states in dairy cows in previous studies (Schmied et al., 2008; Proctor and Carder, 2014). However, we observed predominantly *back up* positions and surprisingly rare occurrences of *ear low*. One possible reason might have been the strokers' position kneeling next to the lying animal and resulting in the auditory signal being located above and behind the heifers' ears in both conditions. Since the ear position pattern was very similar to the one found in our previous study without vocal stimulation (Lange et al., 2020), however, the effect of the auditory stimulus seems not to have had a strong influence on ear positions, possibly because cattle have a relatively low sound-localization acuity compared with other mammals (Heffner and Heffner, 1992); the stroker's position relative to the animal's head may nevertheless be relevant.

Furthermore, the effects that we saw in STIM were not observed in POST, contrary to our hypothesis of longer-lasting effects of the treatment on behavior. However, some of the observed behaviors (such as *neck stretching* and the different ear positions) are more immediate reactions to positive stimuli and do not allow to observe longer-lasting changes in affective states.

### Comparison of the Treatments

As there were no significant differences in the behavioral reactions to the two different auditory stimuli, stroking and talking in a gentle voice *per se* seem to have a stronger effect on the behavior than the source of the auditory stimulus. As this experiment did not include a treatment where the animals were stroked without any auditory stimulation, we cannot infer any information on whether gentle talking in general enhances or diminishes the positive effects of stroking, but the results are very similar to our previous study, where the animals were stroked without acoustic stimulation. Stroking can elicit quite strong effects on physiology and behavior in different species (rats: Holst et al., 2005; cows: Schmied et al., 2010; cats: Gourkow et al., 2014; lambs: Coulon et al., 2015; horses: Lansade et al., 2018), which might exceed possible consequences of small differences in auditory stimuli. Regarding the absence of significant differences in behavior, it seems plausible that the heifers did not discern the two auditory stimuli, at least not to an extent where it would have affected their behavior. Furthermore, the mismatch of experimenter and playback voice did not have a significant effect on any of the behaviors. Indeed, there is a substantial amount of literature in different species indicating that they do not necessarily distinguish playback from live auditory stimuli: playback is used successfully in studies investigating bird behavior (Douglas and Mennill, 2010), dogs react to dog-directed human speech played back from a loudspeaker (Ben-Aderet et al., 2017; Benjamin and Slocombe, 2018), and dairy cows increase their production when exposed to a playback of calf vocalizations (Pollock and Hurnik, 1978; McCowan et al., 2002; no effect if calves are reared with their mothers: Zipp et al., 2013). Other characteristics of speech might thus have a stronger impact on the animals' behavior than the characteristics induced by the type of source.

On the other hand, the analysis of cardiac parameters points toward a different perception of the two auditory stimuli. In both conditions, *HR* increased from PRE to STIM and decreased from STIM to POST, but this decrease was significantly more pronounced in the “live” condition, indicating a stronger relaxation effect of live talking after the presentation of the stimulus. The slight increase of *HR* during STIM in both conditions seems to contradict our expectation that our treatment would induce a low-arousal state. However, it is in line with previous findings reporting an increased *HR* of lying animals that were licked by conspecifics (Laister et al., 2011) or receiving a stroking treatment (Lange et al., 2020) and might be caused by physical reactions to stroking (e.g., *neck stretching*) more than by a meaningful change in arousal or affective state (Lange et al., 2020).

Independently of the changes in *HR*, there were some significant effects of the conditions on HRV parameters: *HF* increased in POST in the “live” condition, but decreased in POST in the “playback” condition. It is widely accepted that *HF* increases with increasing activity of the parasympathetic branch of the autonomic nervous system (Task Force of ESP and NASPE, 1996; von Borell et al., 2007). The increased values suggest a higher parasympathetic activity after stroking in the “live,” but not the “playback” condition. An increased *HF* may be associated with positive emotions (McCraty et al., 1995; von Borell et al., 2007) and was found in horses regularly receiving a relaxing massage (Kowalik et al., 2017). This increase in *HF* was not accompanied by an increase in *RMSSD*, although both represent vagal activity and are often correlated (Task Force of ESP and NASPE, 1996; Hagen et al., 2005; von Borell et al., 2007; Shaffer et al., 2014). However, changes in *RMSSD* were not consistently observed in other studies investigating different affective states in animals (Reefmann et al., 2012; Travain et al., 2016). *RMSSD* might therefore be a suboptimal indicator of animal affective states (Gygax et al., 2013; Tamioso et al., 2018). A different pattern emerged for *SDNN*: values increased from PRE to STIM in the “live” condition, and decreased again in POST, whereas in the “playback” condition, *SDNN* reached its highest values in POST. *SDNN* reflects influences of both parasympathetic and sympathetic activity (von Borell et al., 2007; Shaffer et al., 2014). Together with the decrease of *RMSSD/SDNN* during live talking, these findings might indicate that the “live” condition led to higher sympathetic activity during stroking and talking, possibly indicating positive arousal in response to being stroked (Tamioso et al., 2018). The increase of *RMSSD/SDNN* in “live” in POST is in line with increased values observed in sheep being brushed by a familiar human (Tamioso et al., 2018), and, in combination with the observed increase of *HF* in POST in “live,” indicates a shift toward vagal dominance after live talking. These patterns were not observed in the “playback” condition; contrarily, *SDNN* increased in POST, while *RMSSD/SDNN* and *HF* decreased slightly, possibly indicating a relative shift towards sympathetic regulation after stroking with “playback” stimulation.

In combination, the HRV results suggest that live talking may have been more pleasurable to the animals than “playback” and led to increased parasympathetic activity in the POST phase. They thus support the interpretation of a more pronounced relaxation effect indicated by the stronger decrease of *HR* in POST in “live” than in “playback.” The difference between the two auditory stimuli might be caused by losses of lower and higher frequencies of recorded sound, which have been found to cause a decline in dog's responses to commands, especially in the absence of certain non-verbal cues (Fukuzawa et al., 2005). As we could not measure the actual sound pressure reaching the animals' ears directly, we can neither exclude the possibility that there might have been other systematic differences between the acoustic signals produced by two sources, such as consistent differences in volume, which might have contributed to eliciting higher or lower arousal. Another difference between the situations might have been produced by a subconscious change of the stroker's body language or attention toward the animal during live talking. However, stroker behavior was standardized as far as possible – in both conditions, the stroker was calmly sitting next to the heifer's shoulder, focused on stroking the animal. Great care was taken to match the “playback” condition not only in body posture and calm breathing, but also in mental focus and intention of interacting gently with the animal, trying to minimize possible differences in non-verbal communication.

We hypothesized that the higher degree of standardization in the “playback” stimulus would lead to decreased variability in the data. However, the variability of the responses as indicated by the precision parameters revealed a conflicting pattern, indicating that the relationship between the degree of standardization of the treatment and the variability in the observed behavior is more complex than expected or has different effects on different parameters. The higher degree of standardization in “playback” stimuli did not lead to a generally reduced variability and therefore should not be the main criterion for preference of playback stimuli for gentle human-animal interactions in experimental settings.

## Conclusion

Our experiment leads to the conclusion that gentle stroking in combination with gentle vocal stimulation can induce positive affective states in habituated heifers, both when the experimenter is talking directly to the animal and when the vocal stimulus is played back from a recording. However, changes in cardiac parameters point toward a more positive experience and longer-lasting relaxation effects of live talking. Taking into account the inconclusive results regarding the effects of a higher degree of standardization on the variability of the data, we conclude that the use of recorded auditory stimuli to promote positive affective states in human-animal interactions in experimental settings is possible, but not necessarily preferable.

## Data Availability Statement

The raw data supporting the conclusions of this article will be made available by the authors, without undue reservation.

## Ethics Statement

The animal study was reviewed and approved by Ethics and Animal Welfare Committee of Vetmeduni Vienna (Ethik und Tierschutzkommission), project number ETK-02/04/2017; Veterinärplatz 1, 1210 Vienna.

## Author Contributions

SL and SW: conceptualization. SL, SW, LB, AL, and AF: methodology. AL: formal analysis, visualization, and writing—original draft preparation. AL and LB: investigation. SL, SW, and AL: writing—review and editing. SW: supervision. SL: project administration and funding acquisition. All authors have read and agreed to the published version of the manuscript.

## Conflict of Interest

The authors declare that the research was conducted in the absence of any commercial or financial relationships that could be construed as a potential conflict of interest.
