# Supplementary material for: Talking to Cows: Reactions to Different Auditory Stimuli During Gentle Human-Animal Interactions
Source: Front Psychol. 2020 Oct 15;11:579346. doi: 10.3389/fpsyg.2020.579346 (PMC7593841; doi:10.3389/fpsyg.2020.579346)
Supplement: Supplementary file 1 [file Data_Sheet_1.pdf]

Supplementary Material

1 Supplementary Data

Supplementary Data S1. Auditory stimulus for the ‘playback’ condition containing a sample of Experimenter A talking in a gentle voice in the same way as in the ‘live’ condition, using phrases with positive content (in German) that were spoken calmly, with long low-pitched vowels and a decrease in pitch towards the end of the words or phrases.

2 Supplementary Figures and Tables

2.1 Supplementary Figures

Supplementary Figure S1. Example photographs of ear positions (Lange et al., 2020). The ear postures are described relative to the vertical axis, an imaginary line through the poll and the caudo-ventral edge of the mandible angle, and the horizontal axis, an imaginary line between the bases of the ears. “Back” means the ear is pointing towards the back of the head, “forward” refers to the rostral end of the head, “up” describes the ear pointing dorsally and “down” pointing ventrally. The example photograph for “forward down” was taken in another experiment because the position did not occur in our study.

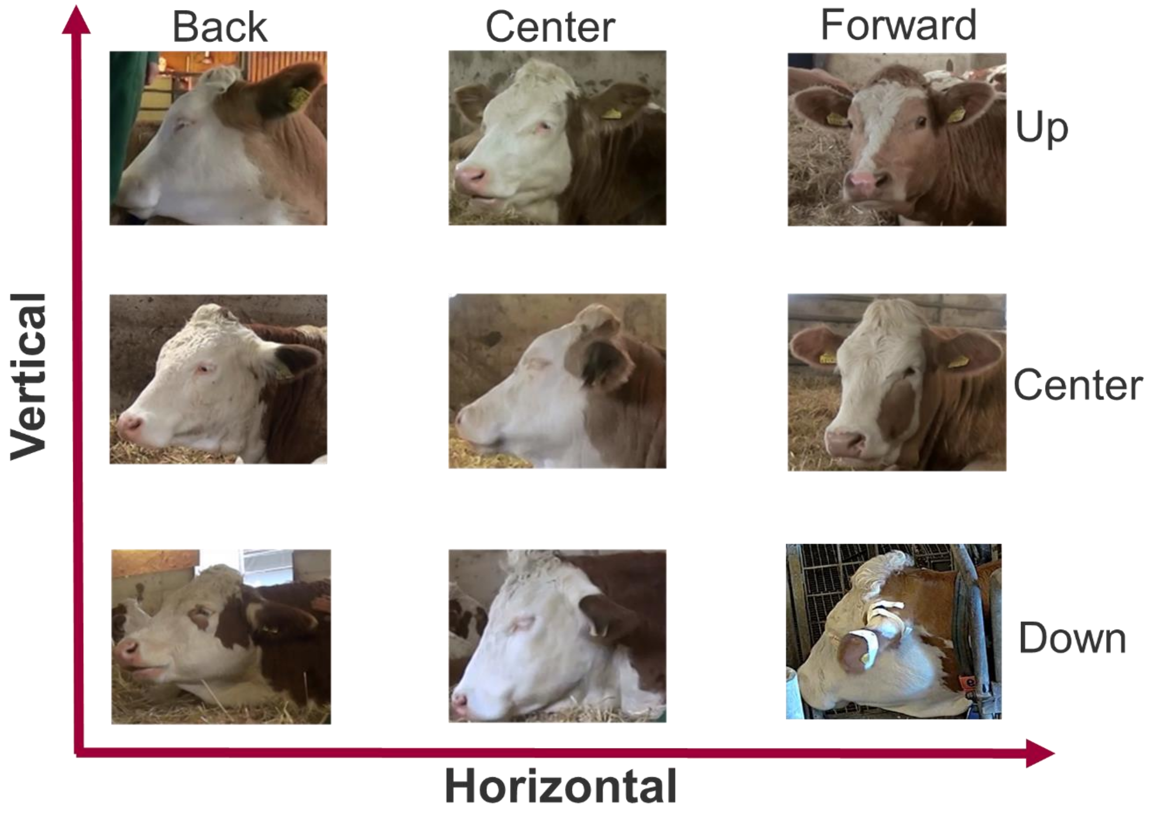

Supplementary Figure S2. Example photographs (Lange et al., 2020) of ear positions with lines indicating (a) the vertical axis (yellow, through the poll and the caudo-ventral edge of the mandible angle) and (b) the horizontal axis (red, between the bases of the ears).

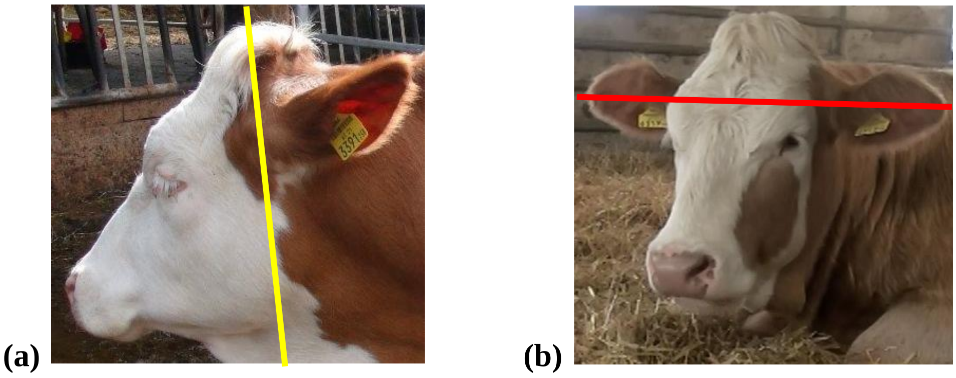

(a) (b)

2.2 Supplementary Tables

Supplementary Table S1. Full and reduced models for the different behaviors of the heifers (n = 28): comparison between the different auditory stimuli over the three phases. Statistically significant results appear in bold. CL: confidence limits. Statistics: GLMMs.

**Full model *neck stretching*[1]**								
**Part**	**Effects**	**Coefficients**	**SE**	**CL_{lower}**	**CL_{upper}**	**z**	**df**	**p**
Mean	(Intercept)	-4.285	0.219	-4.658	-4.008			- [2]
	Treatment (playback)[3]	-0.491	0.256	-0.864	-0.078	-1.923		0.055
	Phase (STIM)[4]	0.698	0.305	0.296	1.157	2.287		0.022
	Phase (POST)[4]	-0.019	0.192	-0.321	0.280	-0.097		0.923
	Test	-0.046	0.068	-0.146	0.051	-0.672		0.502
	Treatment (playback)[3] * Phase (STIM)[4]	0.020	0.264	-0.382	0.419	0.074		0.941
	Treatment (playback)[3] * Phase (POST)[4]	0.011	0.271	-0.412	0.404	0.040		0.968
Precision	(Intercept)	2.924	0.130	2.734	3.231			-
	**Treatment (playback)[3]**	0.674	0.172	0.327	1.022	3.912		**< 0.001**
**Reduced model *neck stretching* without insignificant treatment*phase interaction[1]**								
**Part**	**Effects**	**Coefficients**	**SE**	**CL_{lower}**	**CL_{upper}**	**z**	**df**	**p**
Mean	(Intercept)	-4.291	0.199	-4.577	-4.022			-
	Treatment (playback)[3]	-0.478	0.173	-0.771	-0.236	-2.768		0.006
	**Phase (STIM)[4]**	0.708	0.273	0.317	1.129	**2.594**		**0.009**
	Phase (POST)[4]	-0.013	0.135	-0.223	0.193	-0.097		0.923
	Test	-0.046	0.068	-0.147	0.057	-0.673		0.501
Precision	(Intercept)	2.926	0.128	2.699	3.216			-
	**Treatment (playback)[3]**	0.670	0.165	0.348	1.001	16.177		**< 0.001**
**Full model *contact*[1]**								
**Part**	**Effects**	**Coefficients**	**SE**	**CL_{lower}**	**CL_{upper}**	**z**	**df**	**p**
Mean	(Intercept)	-3.412	0.248	-3.729	-3.166			-
	Treatment (playback)[3]	-0.647	0.346	-1.043	-0.273	-1.868		0.062
	Phase (STIM)[4]	0.156	0.254	-0.121	0.45	0.615		0.539
	Phase (POST)[4]	0.064	0.254	-0.233	0.337	0.251		0.802
	Test	-0.038	0.074	-0.121	0.041	-0.518		0.605
	Treatment (playback) [3] * Phase (STIM)[4]	-0.039	0.357	-0.465	0.359	-0.110		0.912
	Treatment (playback)[3] * Phase (POST)[4]	0.064	0.357	-0.328	0.475	0.179		0.858
Precision	(Intercept)	2.004	0.127	1.827	2.318			-
	**Treatment (playback)[3]**	0.778	0.180	0.438	1.117	**4.321**		**< 0.001**
**Full model *resting head*[1]**								
**Part**	**Effects**	**Coefficients**	**SE**	**CL_{lower}**	**CL_{upper}**	**z**	**df**	**p**
Mean	(Intercept)	-2.669	0.195	-3.018	-2.402			-

	Treatment (playback)(3)	-1.122	0.269	-1.541	-0.682	-4.165		0.000
	Phase (STIM)(4)	0.115	0.202	-0.187	0.424	0.569		0.569
	Phase (POST)(4)	0.105	0.202	-0.192	0.438	0.520		0.603
	Test	-0.042	0.059	-0.125	0.043	-0.718		0.473
	Treatment (playback)(3) * Phase (STIM)(4)	0.050	0.278	-0.379	0.460	0.181		0.857
	Treatment (playback)(3) * Phase (POST)(4)	0.013	0.278	-0.416	0.422	0.048		0.961
Precision	(Intercept)	1.046	0.124	0.857	1.374	8.433		-
	**Treatment (playback)(3)**	1.413	0.178	1.056	1.803	7.948		**< 0.001**
**Full model *flicking***								
**Part**	**Effects**	**Coefficients**	**SE**	**CL_{lower}**	**CL_{upper}**	**χ^2**	**df**	**p**
Mean	(Intercept)	-3.792	0.139	-4.059	-3.539			-
	Treatment (playback)(3)	0.296	0.186	-0.068	0.650			-
	Phase (STIM)(4)	-0.677	0.155	-1.005	-0.385			-
	Phase (POST)(4)	-0.259	0.104	-0.469	-0.051			-
	Test	0.015	0.081	-0.142	0.166	0.036	1	0.849
	Treatment:Phase(5)					0.661	2	0.718
	Treatment (playback)(3) * Phase (STIM)(4)	-0.125	0.168	-0.454	0.235			-
	Treatment (playback)(3) * Phase (POST)(4)	-0.090	0.149	-0.383	0.204			-
Precision	(Intercept)	3.978	0.135	3.754	4.232			-
	Treatment (playback)(3)	-0.177	0.176	-0.506	0.119	1.010	1	0.315
**Reduced model *flicking* without insignificant treatment*phase interaction**								
**Part**	**Effects**	**Coefficients**	**SE**	**CL_{lower}**	**CL_{upper}**	**χ^2**	**df**	**p**
Mean	(Intercept)	-3.720	0.130	-3.960	-3.464			-
	Treatment (playback)(3)	0.166	0.160	-0.153	0.472	1.073	1	0.300
	**Phase (STIM)(4)**	**-0.743**	**0.132**	**-1.008**	**-0.501**	**32.520**	**2**	**< 0.001**
	Phase (POST)(4)	-0.306	0.075	-0.442	-0.143			
	Test	0.018	0.080	-0.145	0.185	0.050	1	0.823
Precision*(6)*								
**Full model *eye closed***								
**Part**	**Effects**	**Coefficients**	**SE**	**CL_{lower}**	**CL_{upper}**	**χ^2**	**df**	**p**
Mean	(Intercept)	-2.033	0.153	-2.338	-1.758			-
	Treatment (playback)(3)	0.162	0.200	-0.238	0.554			-
	Phase (STIM)(4)	0.039	0.162	-0.299	0.35			-
	Phase (POST)(4)	0.304	0.165	-0.011	0.614			-
	Test	-0.012	0.056	-0.120	0.089	0.045	1	0.832
	Treatment:Phase(5)					1.481	2	0.477
	Treatment (playback)(3) * Phase (STIM)(4)	-0.225	0.231	-0.673	0.220			-
	Treatment (playback)(3) * Phase (POST)(4)	-0.260	0.232	-0.695	0.205			-
Precision	(Intercept)	0.617	0.115	0.446	0.874			-
	Treatment (playback)(3)	-0.003	0.150	-0.313	0.285	0.000	1	0.986
**Full model *ear changes***								
**Part**	**Effects**	**Coefficients**	**SE**	**CL_{lower}**	**CL_{upper}**	**χ^2**	**df**	**p**
Mean	(Intercept)	2.530	0.111	2.305	2.737			-
	Treatment (playback)(3)	0.015	0.128	-0.251	0.268			-
	Phase (STIM)(4)	-0.882	0.146	-1.171	-0.601			-
	Phase (POST)(4)	-0.168	0.095	-0.366	0.011			-
	Test	-0.113	0.053	-0.208	-0.001	3.970	1	0.046
	Treatment:Phase(5)					0.030	2	0.985
	Treatment (playback)(3) * Phase (STIM)(4)	0.023	0.143	-0.248	0.34			-
	Treatment (playback)(3) * Phase (POST)(4)	0.018	0.133	-0.229	0.276			-
Precision*(7)*								-
**Reduced model *ear changes* without insignificant treatment*phase interaction**								
**Part**	**Effects**	**Coefficients**	**SE**	**CL_{lower}**	**CL_{upper}**	**χ^2**	**df**	**p**
Mean	(Intercept)	2.524	0.105	2.303	2.728			-
	Treatment (playback)(3)	0.028	0.104	-0.182	0.240	0.070	1	0.791
	**Phase (STIM)(4)**	-0.870	0.128	-1.138	-0.622	31.526	**2**	**< 0.001**
	Phase (POST)(4)	-0.159	0.067	-0.292	-0.035			-

	Test	-0.113	0.053	-0.221	-0.014	3.980	1	0.046
Precision*(7)*								-
**Full model *back up***								
**Part**	**Effects**	**Coefficients**	**SE**	**CL$_{lower}$**	**CL$_{upper}$**	**χ^2**	**df**	**p**
Mean	(Intercept)	0.085	0.194	-0.343	0.400			-
	Treatment (playback)(3)	-0.126	0.201	-0.523	0.262			-
	Phase (STIM)(4)	1.248	0.203	0.833	1.616			-
	Phase (POST)(4)	0.196	0.155	-0.110	0.532			-
	Test	0.208	0.079	0.051	0.353	5.900	1	0.015
	Treatment:Phase(5)					0.666	2	0.717
	Treatment (playback)(3) * Phase (STIM)(4)	-0.175	0.230	-0.614	0.291			-
	Treatment (playback)(3) * Phase (POST)(4)	-0.141	0.224	-0.59	0.327			-
Precision	(Intercept)	0.992	0.111	0.791	1.199			-
	Treatment (playback)(3)	-0.093	0.136	-0.368	0.16	0.469	1	0.493
**Reduced model *back up* without insignificant treatment*phase interaction**								
**Part**	**Effects**	**Coefficients**	**SE**	**CL$_{lower}$**	**CL$_{upper}$**	**χ^2**	**df**	**p**
Mean	(Intercept)	0.135	0.183	-0.270	0.436			-
	Treatment (playback)(3)	-0.227	0.156	-0.517	0.057	2.122	1	0.145
	**Phase (STIM)(4)**	1.160	0.167	0.826	1.445	**30.705**	**2**	**< 0.001**
	Phase (POST)(4)	0.128	0.112	-0.082	0.351			-
	Test	0.207	0.079	0.056	0.359	5.845	1	0.016
Precision	(Intercept)	0.980	0.109	0.778	1.168			-
	Treatment (playback)(3)	-0.073	0.132	-0.339	0.174	0.308	1	0.579
**Full model *back centre***								
**Part**	**Effects**	**Coefficients**	**SE**	**CL$_{lower}$**	**CL$_{upper}$**	**χ^2**	**df**	**p**
Mean	(Intercept)	-2.049	0.152	-2.368	-1.777			-
	Treatment (playback)(3)	0.428	0.190	0.042	0.796			-
	Phase (STIM)(4)	-0.211	0.155	-0.518	0.098			-
	Phase (POST)(4)	0.244	0.154	-0.051	0.571			-
	Test	-0.100	0.052	-0.196	0.002	3.776	1	0.052
	Treatment:Phase(5)					0.113	2	0.945
	Treatment (playback)(3) * Phase (STIM)(4)	-0.008	0.226	-0.458	0.449			-
	Treatment (playback)(3) * Phase (POST)(4)	-0.069	0.225	-0.507	0.377			-
Precision	(Intercept)	1.239	0.120	1.054	1.489			-
	**Treatment (playback)(3)**	-0.461	0.142	-0.740	-0.204	**10.357**	**1**	**0.001**
**Reduced model *back centre* without insignificant treatment*phase interaction**								
**Part**	**Effects**	**Coefficients**	**SE**	**CL$_{lower}$**	**CL$_{upper}$**	**χ^2**	**df**	**p**
Mean	(Intercept)	-2.035	0.138	-2.322	-1.788			-
	Treatment (playback)(3)	0.400	0.137	0.128	0.664	8.029	1	0.052
	**Phase (STIM)(4)**	-0.214	0.113	-0.460	-0.008	**13.500**	**2**	**0.001**
	Phase (POST)(4)	0.212	0.113	-0.013	0.453			-
	Test	-0.099	0.052	-0.203	0.001	3.771	1	0.052
Precision	(Intercept)	1.235	0.119	1.051	1.474			-
	**Treatment (playback)(3)**	-0.457	0.141	-0.717	-0.181	**10.273**	**1**	**0.001**
**Full model *forward up*(1)**								
**Part**	**Effects**	**Coefficients**	**SE**	**CL$_{lower}$**	**CL$_{upper}$**	**z**	**df**	**p**
Mean	(Intercept)	-2.814	0.235	-3.098	-2.549			-
	Treatment (playback)(3)	-0.009	0.337	-0.392	0.363	-0.027		0.978
	Phase (STIM)(4)	-0.293	0.257	-0.588	0.001	-1.139		0.255
	Phase (POST)(4)	-0.011	0.256	-0.318	0.280	-0.041		0.967
	Test	-0.038	0.074	-0.129	0.051	-0.517		0.605
	Treatment (playback)(3) * Phase (STIM)(4)	-0.014	0.366	-0.448	0.415	-0.038		0.970
	Treatment (playback)(3) * Phase (POST)(4)	-0.088	0.365	-0.473	0.339	-0.241		0.810
Precision	(Intercept)	1.661	0.120	1.475	1.971			-
	Treatment (playback)(3)	-0.015	0.173	-0.350	0.328	-0.087		0.930
**Reduced model *forward up* without insignificant treatment*phase interaction(1)**								
**Part**	**Effects**	**Coefficients**	**SE**	**CL$_{lower}$**	**CL$_{upper}$**	**z**	**df**	**p**
Mean	(Intercept)	-2.797	0.157	-3.058	-2.578			-
	Treatment (playback)(3)	-0.044	0.199	-0.368	0.270	-0.219		0.827

	**Phase (STIM)[4]**	-0.300	0.135	-0.503	-0.072	**-2.216**		**0.027**
	Phase (POST)[4]	-0.054	0.135	-0.269	0.164	-0.401		0.689
	Test	-0.038	0.055	-0.123	0.049	-0.700		0.484
Precision	(Intercept)	1.660	0.120	1.474	1.932			-
	Treatment (playback)[3]	-0.014	0.172	-0.334	0.339	-0.083		0.934
**Full model *ear low***								
**Part**	**Effects**	**Coefficients**	**SE**	**CL_{lower}**	**CL_{upper}**	**χ^2**	**df**	**p**
Mean	(Intercept)	-0.463	0.444	-1.307	0.312			-
	Treatment (playback)[3]	0.023	0.461	-0.913	0.892			-
	Phase (STIM)[4]	-1.138	0.434	-2.009	-0.350			-
	Phase (POST)[4]	0.409	0.407	-0.341	1.144			-
	Test	-0.433	0.178	-0.801	-0.087	5.356	1	0.052
	Treatment:Phase[5]					0.664	2	0.717
	Treatment (playback)[3] * Phase (STIM)[4]	0.307	0.592	-0.805	1.496			-
	Treatment (playback)[3] * Phase (POST)[4]	-0.172	0.568	-1.247	0.917			-
Precision[7]								-
**Reduced model *ear low* without insignificant treatment*phase interaction**								
**Part**	**Effects**	**Coefficients**	**SE**	**CL_{lower}**	**CL_{upper}**	**χ^2**	**df**	**p**
Mean	(Intercept)	-0.477	0.411	-1.339	0.306			-
	Treatment (playback)[3]	0.055	0.325	-0.568	0.666	0.028	1	0.052
	**Phase (STIM)[4]**	-0.977	0.303	-1.569	-0.374	**19.094**	**2**	**0.000**
	Phase (POST)[4]	0.321	0.284	-0.213	0.861			-
	Test	-0.429	0.177	-0.782	-0.123	5.286	1	0.021
Precision[7]								-

(1) overdispersion; SE, z- and p-values corrected for overdispersion (p-value based on Wald's z-approximation; recognizable by no df indicated)
(2) not shown because of having a very limited interpretation
(3) dummy coded ('live' as reference category)
(4) dummy coded (PRE as reference category)
(5) the indicated test refers to the overall effect of the interaction between treatment and phase
(6) no precision part available due to convergence problems
(7) no precision part because the model is not based on beta error structure

Supplementary Table S2. Full models for HR(V) parameters of the heifers (n = 26): comparison between the different auditory stimuli over the three phases. Statistically significant results appear in bold. CL: confidence limits. Statistics: LMMs.

**Full model *HR***							
**Effects**	**Coefficients**	**SE**	**CL_{lower}**	**CL_{upper}**	**χ^2**	**df**	**p**
(Intercept)	74.081	2.169	69.884	78.262			-[1]
Treatment (playback)[2]	-2.364	2.111	-6.543	2.116			-
Phase (STIM)[3]	1.541	0.401	0.815	2.305			-
Phase (POST)[3]	-0.555	0.352	-1.234	0.136			-
Rumination	1.694	0.215	1.262	2.106	43.959	1	0.000
sin(rad.time)1	-2.239	1.046	-4.508	0.300	2.799	1	0.094
cos(rad.time)1	-8.550	1.102	-11.054	-5.957	11.193	1	0.001
Age	-4.112	1.982	-8.466	-0.087	12.200	1	0.000
Group	-10.326	3.360	-17.398	-2.942	5.786	1	0.016
**Treatment:Phase[4]**					**9.917**	**2**	**0.007**
Treatment (playback) [2] * Phase (STIM)[3]	-0.245	0.448	-1.181	0.600			-
Treatment (playback) [2] * Phase (POST)[3]	1.111	0.449	0.194	1.959			-
**Full model *RMSSD***							
**Effects**	**Coefficients**	**SE**	**CL_{lower}**	**CL_{upper}**	**χ^2**	**df**	**p**
(Intercept)	2.808	0.145	2.512	3.130			-[1]
Treatment (playback)[2]	-0.067	0.077	-0.229	0.083			-
Phase (STIM)[3]	-0.021	0.054	-0.131	0.095			-
Phase (POST)[3]	0.020	0.056	-0.097	0.132			-
Heart rate	-0.349	0.055	-0.471	-0.229	22.351	1	0.000
Rumination	0.047	0.028	-0.012	0.108	1.813	1	0.178
sin(rad.time)1	-0.032	0.076	-0.210	0.155	0.112	1	0.738
cos(rad.time)1	-0.062	0.099	-0.293	0.161	0.64	1	0.424
Age	-0.095	0.129	-0.383	0.182	0.414	1	0.520

Group	-0.258	0.278	-0.879	0.355	1.174	1	0.279
Treatment:Phase[4]					0.673	2	0.714
Treatment (playback)[2] * Phase (STIM)[3]	0.013	0.072	-0.122	0.148			-
Treatment (playback)[2] * Phase (POST)[3]	0.057	0.073	-0.082	0.209			-
**Full model *SDNN***							
**Effects**	**Coefficients**	**SE**	**CL_{lower}**	**CL_{upper}**	**χ^2**	**df**	**p**
(Intercept)	5.208	0.291	3.030	3.452			-[1]
Treatment (playback)[2]	-0.046	0.245	-0.212	0.135			-
Phase (STIM)[3]	0.447	0.208	0.039	0.335			-
Phase (POST)[3]	0.088	0.207	-0.100	0.186			-
Heart rate	-0.562	0.134	-0.345	-0.117	11.671	1	0.001
Rumination	-0.098	0.087	-0.091	0.034	1.486	1	0.223
sin(rad.time)1	-0.142	0.220	-0.225	0.097	2.608	1	0.456
cos(rad.time)1	-0.501	0.270	-0.426	-0.041	4.173	1	0.243
Age	-0.173	0.203	-0.254	0.064	0.328	1	0.567
Group	-0.389	0.442	-0.511	0.185	1.15	1	0.284
**Treatment:Phase[4]**					**9.963**	**2**	**0.007**
Treatment (playback)[2] * Phase (STIM)[3]	-0.466	0.283	-0.346	0.029			-
Treatment (playback)[2] * Phase (POST)[3]	0.411	0.284	-0.053	0.317			-
**Full model *RMSSD/SDNN***							
**Effects**	**Coefficients**	**SE**	**CL_{lower}**	**CL_{upper}**	**χ^2**	**df**	**p**
(Intercept)	0.821	0.040	0.737	0.906			-[1]
Treatment (playback)[2]	-0.036	0.026	-0.085	0.018			-
Phase (STIM)[3]	-0.063	0.023	-0.106	-0.016			-
Phase (POST)[3]	-0.005	0.024	-0.053	0.039			-
Heart rate	-0.093	0.016	-0.126	-0.057	27.486	1	0.000
Rumination	0.023	0.009	0.003	0.043	4.815	1	0.028
sin(rad.time)1	-0.006	0.023	-0.055	0.040	-0.01	1	1
cos(rad.time)1	0.034	0.028	-0.034	0.093	1.107	1	0.293
Age	-0.048	0.030	-0.118	0.022	1.699	1	0.192
Group	-0.032	0.066	-0.181	0.119	0.173	1	0.677
**Treatment:Phase[4]**					**8.378**	**2**	**0.015**
Treatment (playback)[2] * Phase (STIM)[3]	0.063	0.030	0.002	0.117			-
Treatment (playback)[2] * Phase (POST)[3]	-0.022	0.03	-0.083	0.038			-
**Full model *HF***							
**Effects**	**Coefficients**	**SE**	**CL_{lower}**	**CL_{upper}**	**χ^2**	**df**	**p**
(Intercept)	2.903	0.157	2.559	3.219			-[1]
Treatment (playback)[2]	-0.007	0.119	-0.241	0.237			-
Phase (STIM)[3]	-0.043	0.114	-0.256	0.187			-
Phase (POST)[3]	0.222	0.107	0.000	0.435			-
Heart rate	-0.584	0.081	-0.765	-0.407	41.484	1	0.000
Rumination	0.085	0.044	-0.006	0.177	2.994	1	0.084
sin(rad.time)1	0.020	0.098	-0.198	0.214	0.038	1	0.846
cos(rad.time)1	0.328	0.115	0.064	0.581	6.801	1	0.009
Age	-0.220	0.135	-0.513	0.065	2.226	1	0.136
Group	-0.153	0.315	-0.806	0.576	0.206	1	0.650
**Treatment:Phase[4]**					**7.657**	**2**	**0.022**
Treatment (playback)[2] * Phase (STIM)[3]	0.070	0.143	-0.220	0.351			-
Treatment (playback)[2] * Phase (POST)[3]	-0.307	0.143	-0.586	-0.031			-
**Full model *LF***							
**Effects**	**Coefficients**	**SE**	**CL_{lower}**	**CL_{upper}**	**χ^2**	**df**	**p**
(Intercept)	67.999	3.984	60.143	76.398			-[1]
Treatment (playback)[2]	0.081	3.222	-6.357	6.435			-
Phase (STIM)[3]	-0.041	3.074	-5.867	5.811			-
Phase (POST)[3]	-1.584	3.172	-8.046	4.931			-
Heart rate	7.639	1.743	4.001	11.392	15.572	1	0.000
Rumination	1.252	1.185	-1.224	3.678	0.990	1	0.320
sin(rad.time)1	1.013	2.858	-4.777	6.805	0.180	1	0.671
cos(rad.time)1	-4.792	3.523	-12.146	2.750	1.336	1	0.248
Age	0.895	2.997	-5.524	7.294	0.154	1	0.695
Group	5.955	6.842	-8.596	20.433	0.613	1	0.434
Treatment:Phase[4]					0.009	2	0.995
Treatment (playback)[2] * Phase (STIM)[3]	-0.372	4.189	-8.479	7.639			-
Treatment (playback)[2] * Phase (POST)[3]	-0.072	4.210	-7.890	8.384			-

Full model *LF/HF*							
**Effects**	**Coefficients**	**SE**	**CL_{lower}**	**CL_{upper}**	**χ^2**	**df**	**p**
(Intercept)	1.204	0.209	0.747	1.631			-(1)
Treatment (playback)(2)	-0.025	0.164	-0.340	0.295			-
Phase (STIM)(3)	0.005	0.160	-0.297	0.304			-
Phase (POST)(3)	-0.270	0.152	-0.567	0.052			-
Heart rate	0.703	0.102	0.475	0.921	32.913	1	0.000
Rumination	-0.040	0.062	-0.167	0.098	0.373	1	0.542
sin(rad.time)1	-0.028	0.146	-0.327	0.267	-1.902	1	1
cos(rad.time)1	-0.497	0.173	-0.877	-0.146	5.543	1	0.019
Age	0.033	0.169	-0.347	0.376	0.030	1	0.863
Group	0.580	0.404	-0.306	1.429	-1.310	1	1
Treatment:Phase(4)					1.994	2	0.369
Treatment (playback)(2) * Phase (STIM)(3)	-0.024	0.206	-0.408	0.399			-
Treatment (playback)(2) * Phase (POST)(3)	0.337	0.206	-0.068	0.722			-

(1) not shown because of having a very limited interpretation
(2) dummy coded ('live' as reference category)
(3) dummy coded (PRE as reference category)
(4) the indicated test refers to the overall effect of the interaction between treatment and phase
